# Development of Dextran-Coated Magnetic Nanoparticles Loaded with Protocatechuic Acid for Vascular Inflammation Therapy

**DOI:** 10.3390/pharmaceutics13091414

**Published:** 2021-09-07

**Authors:** Maria Anghelache, Mihaela Turtoi, Anca Roxana Petrovici, Adrian Fifere, Mariana Pinteala, Manuela Calin

**Affiliations:** 1 “Medical and Pharmaceutical Bionanotechnologies” Laboratory, Institute of Cellular Biology and Pathology “Nicolae Simionescu” of the Romanian Academy, B.P. Hasdeu 8, 050568 Bucharest, Romania; maria.anghelache@icbp.ro (M.A.); mihaela.carnuta@icbp.ro (M.T.); 2 “Centre of Advanced Research in Bionanoconjugates and Biopolymers” Department, “Petru Poni” Institute of Macromolecular Chemistry, 41A Grigore Ghica-Voda Alley, 700487 Iasi, Romania; petrovici.anca@icmpp.ro (A.R.P.); pinteala@icmpp.ro (M.P.)

**Keywords:** magnetic nanoparticles, dextran, protocatechuic acid, anti-inflammatory activity, endothelial cells, macrophages

## Abstract

Vascular inflammation plays a crucial role in the progression of various pathologies, including atherosclerosis (AS), and thus it has become an attractive therapeutic target. The protocatechuic acid (PCA), one of the main metabolites of complex polyphenols, is endowed with anti-inflammatory activity, but its formulation into nanocarriers may increase its bioavailability. In this study, we developed and characterized dextran shell‒iron oxide core nanoparticles loaded with PCA (MNP-Dex/PCA) and assessed their cytotoxicity and anti-inflammatory potential on cells acting as key players in the onset and progression of AS, namely, endothelial cells (EC) and monocytes/macrophages. The results showed that MNP-Dex/PCA exert an anti-inflammatory activity at non-cytotoxic and therapeutically relevant concentrations of PCA (350 μM) as supported by the reduced levels of inflammatory molecules such as MCP-1, IL-1β, TNF-α, IL-6, and CCR2 in activated EC and M1-type macrophages and functional monocyte adhesion assay. The anti-inflammatory effect of MNP-Dex/PCA was associated with the reduction in the levels of ERK1/2 and p38-α mitogen-activated protein kinases (MAPKs) and NF-kB transcription factor. Our data support the further development of dextran shell-magnetic core nanoparticles as theranostic nanoparticles for guidance, imaging, and therapy of vascular inflammation using PCA or other anti-inflammatory compounds.

## 1. Introduction

Cardiovascular diseases (CVD) are the leading cause of global mortality and contribute significantly to a reduction in quality of life [1]. Until recently, cholesterol accumulation in the arterial wall were considered the primary cause of atherosclerosis (AS) [2]. Both preclinical and clinical research have shown that chronic inflammatory processes and lipid abnormalities interact to cause AS [3]. Although vascular endothelial cells (EC) normally impede the adhesion of circulating immune cells [4], inflammation and hyperlipidemia can stimulate the endothelial expression of cell adhesion molecules, and increase the permeability of blood vessels walls, as well as the neutrophil and monocyte recruitment with the subsequent overproduction of different inflammatory molecules [5]. Many immune cells participate in AS development, but monocyte-derived macrophages play a central role by phagocytosing lipids, which triggers their transformation into foam cells [6]. As chronic inflammation underlies the emergence and evolution of various pathologies, it has become an attractive therapeutic target [7]. The primary signaling molecules regulating chronic inflammatory responses within EC and macrophages are Mitogen-Activated Protein Kinases (MAPKs) and Nuclear Factor-kappa B (NF-kB) [8]. MAPKs are a family of enzymes, more specifically, serine/threonine protein kinases, implicated in the synthesis regulation of several transcription factors that mediate the inflammation at a transcriptional level [9]. Similarly, the transcription factor NF-kB is considered a master regulator of inflammation, which, once activated in response to different types of stimuli, including inflammatory signals, leads to the production of pro-inflammatory cytokines such as interleukin (IL)-1β, IL-6 and TNF-α, and chemokines such as monocyte chemoattractant protein (MCP)-1 [10].

The use of natural bioactive compounds, including polyphenols, in the development of new anti-inflammatory therapies has become an emerging trend in recent years [11]. Although documented evidence exists of polyphenols exhibiting anti-inflammatory activities, their reduced bioavailability and stability represent a major drawback that restricts their use as therapeutic agents [12]. Since nanoparticles can achieve controlled drug targeting and release, a nanotherapeutic approach can overcome these obstacles, as nanotherapy was successfully introduced in cancer treatment and has recently gained prominence in CVD therapeutic strategies [13,14]. Magnetic nanoparticles (MNP), specifically iron oxide nanoparticles, have been widely used for diagnostic purposes, for instance, for visualization of tumors and metastases in the liver, spleen, and lymph nodes, as well as inflammatory lesions, such as atherosclerotic plaques [15]. Based on their magnetic properties, MNP have been extensively studied in the last decade for their potential use in drug targeting [16,17]. The effects of plain MNP on EC are still an area of research that is somewhat underdeveloped. Considering their importance in regulating immune responses and their location at the interface between blood and the vessel wall, more knowledge of MNP’s intrinsic activity on EC will improve theranostic applications [18]. Protocatechuic acid (3,4-dihydroxybenzoic acid; PCA), a phenolic acid found in medicinal plants such as Roselle (*Hibiscus sabdariffa* L.) or Japanese ginkgo (*Ginkgo biloba* L.), has several therapeutic effects associated with anti-oxidant, anti-bacterial, anti-aging, anti-fibrotic, and anti-inflammatory activity, or even anti-cancer properties at the appropriate concentrations [19]. Human studies reported that PCA, produced as a degradation metabolite by the intestinal microbiota from flavonoids such as anthocyanins or procyanidins, is bioavailable in serum after flavonoids intake [20]. After absorption through the intestinal epithelium, PCA may reach systemic blood circulation, delivered to the liver, where it may undergo structural modifications resulting in sulfated and glucuronidated forms that can be distributed to the tissues or excreted in the urine or feces. However, the available concentrations of PCA and its conjugates may be too low to exert their therapeutic benefit at a defined place. To achieve efficient anti-inflammatory activity, PCA has to be concentrated at the inflammatory sites, and intracellular delivery has to be promoted.

With this in mind, we aimed to develop and characterize core-shell magnetic nanoparticles covered with dextran (MNP-Dex) and loaded with protocatechuic acid (MNP-Dex/PCA), as well as to assess their cytotoxicity and anti-inflammatory potential on cells playing a central role in the onset and progression of AS, namely, EC and macrophages. The anti-inflammatory activity of MNP-Dex/PCA was investigated by assessing the adhesion capacity of THP-1 monocytes to tumor necrosis factor (TNF)-α activated EC (EA.hy926 cells) and treated with MNP-Dex/PCA. In addition, the effect of MNP-Dex/PCA on the protein expression of MAPKs ERK1/2 and p38-α isoform, as well as the transcription factor NF-kB and levels of MCP-1 chemokine in activated EC and pro-inflammatory factors IL-1β, TNF-α, IL-6, and CCR2 in THP-1-derived M1 type (inflammatory) macrophages, were measured.

To the best of our knowledge, this is the first study showing that core-shell magnetic nanoparticles covered with dextran and loaded with PCA have an anti-inflammatory activity at non-cytotoxic concentrations by reducing the levels of inflammatory molecules such as MCP-1, IL-1β, TNF-α, IL-6, and CCR2 in activated EC and M1-type inflammatory macrophages. The anti-inflammatory effect of MNP-Dex/PCA was associated with diminution in the levels of MAPKs, ERK1/2, p38-α, and NF-kB transcription factor.

The data support the use and further development of dextran-coated MNP loaded with PCA as a new theranostic system for guidance and therapeutic action of PCA in various chronic inflammatory illnesses, including AS. The formulation of PCA into magnetic nanoparticles may assure a targeted delivery at the affected site, due to the magnetic properties of the nanocarrier, and the augmented efficiency of PCA as an anti-inflammatory agent by improving its cellular internalization.

## 2. Materials and Methods

### 2.1. Materials

Commercial sources of the primary reagents and consumables used in this study were as follows: ferric chloride (FeCl_3_·6H_2_O), ferrous chloride (FeCl_2_·4H_2_O), 25% ammonium solution (25%), PCA, ammonium persulfate (APS), 2,7-bis(2-carboxyethyl)-5(6)-carboxyfluorescein acetoxymethyl ester (BCECF-AM), dexamethasone (Dexa), Dulbecco’s modified Eagle’s medium, ethylenediaminetetraacetic acid tetrasodium salt dihydrate (EDTA), glycerol, hydrochloric acid (HCl), lipopolysaccharides from Escherichia coli serotype O111:B, 2-mercaptoethanol, *N*,*N*,*N*′,*N*′-Tetramethyl ethylenediamine (TEMED), phenylmethylsulphonyl fluoride (PMSF), paraformaldehyde (PFA), Ponceau S solution, potassium hexacyanoferrate(II) trihydrate, RPMI-1640 medium, sodium dodecyl sulphate (SDS), Triton-X-100, and Tris-HCl, which were purchased from SIGMA-Aldrich (Merck KGaA, Darmstadt, Germany); fetal bovine serum and penicillin/streptomycin, which were purchased from Thermo Fisher Scientific (Waltham, MA, USA); recombinant human tumor necrosis factor-alpha (TNF-α) from R&D Systems, Inc. (Abingdon, UK); cell culture dishes, which were purchased from TPP^®^ (Trasadingen, Switzerland); the transparent/black 96-well micro test plates, F-bottom, which were purchased from Ratiolab (Ratiolab GmbH, Dreieich, Germany); and UV 96-well micro test plates, F-bottom, which were purchased from Corning Inc. (New York, NY, USA). Dextran was biosynthesized as exopolysaccharides from *Weissella confusa*, a yogurt-isolated lactic acid bacterium, as previously reported [20]. A culture medium consisting of DeMan-Rogosa-Sharpe (MRS) agar (55.3 g/L) and sucrose (80 g/L) dissolved in UHT milk was inoculated with *Weissella confusa*, and after 48 h of fermentation, the dextran was extracted and purified as detailed in [21].

### 2.2. Loading of PCA on Magnetic Nanoparticles

#### 2.2.1. MNP Synthesis

The MNP were synthesized by the coprecipitation method and coated with dextran, as reported previously [22]. Briefly, a solution of 0.871 g FeCl_2_·4H_2_O and 2.23 g FeCl_3_·6H_2_O dissolved in 40 mL of deionized water was deoxygenated by bubbling with nitrogen and heated to 70 °C with mechanical stirring. The pH of the solution was raised to 14 by the dropwise addition of 10 mL of ammonium hydroxide solution (30% NH_4_OH), under a stirring rate of 1000 rpm. The reaction was maintained at room temperature under nitrogen for 30 min, after which the black precipitate formed was extracted by magnetic decantation and washed by centrifugation until it reached neutral pH. MNP were stored in alcohol at 4 °C in the refrigerator and washed followed by redispersion into water before use.

#### 2.2.2. Synthesis of Dextran-Coated MNP (MNP-Dex)

MNP were coated with dextran by a method reported in our previous work [22]. A solution formed by suspending 100 mg MNP in 50 mL of 2% dextran solution was heated at 80 °C for 3 h under vigorous mechanical stirring. The nanoparticles were magnetically decanted and washed five times with deionized water to remove free polymers.

#### 2.2.3. Synthesis of PCA-Loaded Dextran-Coated MNP (MNP-Dex/PCA)

MNP-Dex were loaded with PCA by our previously reported method applied for polyethyleneimine-coated MNP with minor modifications [23]. Over 1 mL of a 10 mg/mL PCA solution was added to 1 mL of MNP-Dex solution (10 mg/mL) in water and sonicated for 3 min. The aqueous formulations were further mixed and shaken for 20 min at 23 °C, 900 rpm. Subsequently, the PCA-functionalized nanoparticles were separated from the final mixture by centrifugation at 10,000 rpm, for 15 min, followed by two washing steps and redispersion in sterile water.

### 2.3. Physico-Chemical Characterization of PCA-Loaded Magnetic Nanoparticles

#### 2.3.1. Morphological Analysis of MNP-Dex/PCA

The MNP, MNP-Dex and MNP-Dex/PCA dispersed in water were deposited on carbon-coated copper grids (300-mesh sizes) and further investigated by transmission electron microscopy (TEM) using a Hitachi High-Tech HT7700 Transmission Electron Microscope (Hitachi High-Technologies Corporation, Tokyo, Japan) operated at a 100 kV accelerating voltage in high-contrast fashion.

#### 2.3.2. Size and Zeta Potential

The hydrodynamic diameter and Zeta potential of water-dispersed MNP, MNP-Dex, and MNP-Dex/PCA were evaluated at room temperature using the Delsa Nano C Submicron Particle Size Analyzer (Beckman Coulter, Inc., Fullerton, CA, USA) equipped with a laser operating at 658 nm as previously described [23].

#### 2.3.3. PCA Loading on MNP-Dex

The MNP-Dex/PCA suspensions were decanted by centrifugation; a volume of 10 µL of the supernatant was injected into a Zorbax SB-C8 column (5 µm, 150 mm × 4.6 mm), and the volume of the mobile phase was 1.2 mL/min. The mobile gradient phase consisted of A: 0.1% HCOOH in water (pH = 2), and B: acetonitrile: H_2_O at a ratio of 95:5.

Using the PCA calibration curve, the loading efficiency of PCA on MNP-Dex was calculated with the formula:

$$\mathrm{Loading}\;\mathrm{efficiency}\;\left(\%\right)=\frac{\mathrm{total}\;\mathrm{amount}\;\mathrm{of}\;\mathrm{PCA}\;-\;\mathrm{free}\;\mathrm{amount}\;\mathrm{of}\;\mathrm{PCA}}{\mathrm{total}\;\mathrm{amount}\;\mathrm{of}\;\mathrm{PCA}}$$

where the total amount of PCA is the quantity of PCA used for sample preparation, and the free amount of PCA is the amount of PCA determined from the supernatant resulting from the centrifugation of the nanoparticle suspension.

### 2.4. In Vitro Evaluation of PCA-Loaded Magnetic Nanoparticles

#### 2.4.1. Cell Culture

The cell types used for the in vitro studies are human endothelial cell (EC) line EA.hy926 and the human monocytic cell line THP-1, purchased from American Type Culture Collection (ATCC), (Manassas, VA, USA). EC were grown as monolayers in T-25 culture flasks (TPP^®^ Tissue Culture Plates, Trasadingen, Switzerland), while monocytes were cultured in 60 mm low-adherent cell culture dishes (Eppendorf^®^, Hamburg, Germany). Both cell types were maintained under standard culture conditions (37 °C, 95% humidified atmosphere and 5% CO_2_) in Dulbecco’s Modified Eagle Medium (DMEM) with 0.1% glucose or RPMI-1640 medium, respectively, supplemented with 10% fetal bovine serum (FBS) (heat-inactivated in the case of monocytes) and 1% antibiotic solution containing a mixture of penicillin and streptomycin (100 units/mL each). The cultures were periodically sub-cultivated using a Trypsin/EDTA solution, according to the ATCC protocols. The anti-inflammatory effect of MNP-Dex/PCA was assessed on EA.hy926 and M1-polarized THP-1-derived macrophages. To induce monocyte-to-macrophage differentiation, THP-1 cells were cultured for 48 h in the presence of 100 nM phorbol 12-myristate 13-acetate (PMA), obtaining resting macrophages (M0). Next, M0 macrophages were further incubated for 24 h with 20 ng/mL lipopolysaccharides (LPS) to obtain M1-macrophages with a pro-inflammatory phenotype.

#### 2.4.2. Cell Cytotoxicity Assays

##### Colorimetric Assay

The cytotoxicity of different concentrations of MNP-Dex/PCA, MNP-Dex, and free PCA was assessed by the XTT assay as previously described [24]. Briefly, EC were seeded for 24 h at a density of 1.5 × 10^5^ cells/cm^2^ on flat-bottom 96-well plates and incubated for an additional 24 h with increasing concentrations of MNP-Dex/PCA (~1.8‒80 μg/mL nanoparticles corresponding to 10 ÷ 450 μM of loaded PCA). Unfunctionalized nanoparticles (MNP-Dex) and free PCA were used as controls at the corresponding concentrations. At the end of the incubation period, a mixture of XTT reagent and phenazine methosulfate (PMS) was added to each well for 2 h at 37 °C. Next, the absorbance was measured at a wavelength of 450 nm using the Tecan Infinite M200Pro Spectrophotometer microplate reader. Cytotoxicity was normalized to untreated EC (control) and expressed as fold change relative to control.

##### Bioluminescent Assay

The cytotoxicity of various concentrations of MNP-Dex/PCA, as well as of plain MNP-Dex and PCA, was assessed on M1 macrophages after 24 and 48 h and EC after 48 h of incubation using the ToxiLightTM BioAssay Kit (Lonza Bioscience, Basel, Switzerland) [25]. The release of adenylate kinase in the cell medium indicates a loss of cell integrity, which can be quantified by bioluminescent detection. To remove possible interference from uninternalized MNP and MNP-Dex/PCA, supernatants were centrifuged at 10,000 rpm for 10 min. Measurements were made with Mithras LB 940 Multimode Microplate Reader (Berthold Technologies, Oak Ridge, TN, USA) at 1-s integrated reading, as per the manufacturer’s instructions. Cell cytotoxicity was normalized to control cells (M0 macrophages and untreated EC), and results were expressed as fold-change relative to M0 and EC, respectively.

#### 2.4.3. Magnetic Nanoparticles Internalization

The uptake of magnetic nanoparticles was assessed by Prussian Blue staining. Both cell types were cultured at a density of 0.3 × 10^5^ cells/cm^2^ in 48-well plates. EA.hy926 cells were left to adhere for 24 h, after which they were stimulated for another 24 h with 20 ng/mL TNF-α. To evaluate the internalization of magnetic nanoparticles by M1 macrophages, THP-1 cells were activated to macrophages as above mentioned. Both cell types were treated for 24 h with magnetic nanoparticles at an iron concentration of 62 μg/mL, representing the iron content found in MNP-Dex/PCA for 350 µM PCA loaded onto the system. Cells were washed with PBS, fixed in 4% paraformaldehyde for 30 min, and incubated for another 15 min with a fresh mixture of 10% potassium ferricyanide (K_3_[Fe(CN)_6_]) and 20% HCl (1:1 volume ratio) at room temperature. As a result of iron oxide dissolution by hydrochloric acid treatment, ferric ions were released from MNP, forming a water-insoluble complex with potassium ferrocyanide, known as Prussian Blue. Therefore, the cellular compartments containing MNP were stained in a dark blue-purple color [26]. The cytoplasm was counterstained at RT for 10 min with eosin. Bright-field microscopy (Olympus CKX41 inverted microscope) was used to examine the cells, with eight areas per well being acquired and processed using ImageJ software version 1.8.0 (National Institutes of Health (NIH), Bethesda, MD, USA).

#### 2.4.4. Monocyte Adhesion Assay

The functional role of MNP-Dex/PCA in reducing endothelium inflammation was investigated by monocyte adhesion assay, performed as previously reported [27]. EA.hy926 cells were cultured in 24-well plates at a density of 0.5 × 10^5^ cells/cm^2^ and stimulated with 20 ng/mL TNF-α. After 18 h, the media was replaced and the cells were incubated for 24 h with MNP-Dex/PCA at an iron concentration of 62 μg/mL, corresponding to 350 μM of polyphenol loaded on the surface of MNP-Dex nanoparticles. Plain MNP-Dex and free PCA were used as controls at the appropriate concentrations. The monocytes were fluorescently labelled with 1 μg of BCECF-AM per 106 cells and incubated at 2:1 ratio for 30 min with EC that were previously treated with MNP-Dex/PCA, MNP-Dex, and free PCA at 37 °C in RPMI 1640 medium containing 0.05% FBS. After the incubation period, non-adherent monocytes were removed by washing (three times) with warm PBS 1×. The adherent monocytes were examined using an Olympus IX81 (Olympus Corporation, Tokyo, Japan) inverted microscope with a 10× objective and FITC filter, and four areas were acquired per well, which were later processed with ImageJ software.

#### 2.4.5. Immunological Detection of Proteins Involved in the Inflammatory Process

##### Immunoblotting Assay

In order to evaluate the protein expression of the different key molecules involved in the inflammatory response, EA.hy926 and THP-1 cells were seeded in 6-well culture plates at a density of 0.35 × 10^5^ cells/cm^2^ and activated accordingly: EC were stimulated with 20 ng/mL TNF-α, while monocyte-to-M1 phenotype macrophage activation was achieved using 100 nM PMA and 20 ng/mL LPS. Both cell types were incubated for 48 h with MNP-Dex/PCA at a concentration corresponding to 350 μM of polyphenol captured on the surface of magnetic nanoparticles, plain MNP-Dex (62 μg/mL) and PCA (350 μM), as well as dexamethasone (Dexa, 2.5 μM). After the incubation period, cells were lysed in radio immune-precipitation assay (RIPA) buffer [28] and subjected to total protein concentration determination by the bicinchoninic acid protein assay (BCA). The protein extracts were loaded at 30 μg/lane and separated by 5–20% SDS-PAGE, followed by transfer onto a 0.45 μm pore diameter nitrocellulose membrane using the Trans-Blot Semi-Dry system. In order to detect proteins of interest, the following primary antibodies were used for the membrane probing: rabbit anti-p38-α (1:1000, R&D Systems, cat. no. AF8691), mouse anti-ERK1/2 (1:1000, Abcam, cat. no. ab36991), rabbit anti-Bax (1:1000, Thermo Fisher Scientific cat. no. MA5-32031), rabbit anti-NF-κB (1:1000, Abcam, cat. no. ab16502), rabbit anti-MCP-1 (1:2500, Abcam, cat. no. ab9669), rabbit anti-IL-1β (1:1000, Abcam, cat. no. ab9722), rabbit anti-TNF-α (1:1000, Abcam, cat. no. ab215188), mouse anti-CCR2 (1:600, R&D Systems, cat. no. MAB150), and mouse anti-β actin (1:2000, BIO-RAD cat. no. MCA5775GA). This step was carried out overnight at 4 °C, under constant agitation. After washing three times with saline-Tris buffer (TBS), to which 0.1% Tween 20 detergent (TBS-T) was added, the membranes were incubated for 1 h at room temperature (RT) with the corresponding secondary antibody, goat anti-rabbit IgG, or goat anti-mouse IgG conjugated with horseradish peroxidase (HRP, 1:5000, Thermo Fisher Scientific, Waltham, MA, USA, cat. no. 32460 and 32430, respectively). Immunological detection was achieved by using SuperSignalTM West Dura chemiluminescent substrate (Thermo Fisher Scientific cat. no. 34076) and G: BOX Chemi XX6 image analyzer. Band densitometry corresponding to the proteins of interest was performed using the TotalLab TL120 (v2009) software (Nonlinear Dynamics Ltd., Durham, NC, USA), and the values obtained were subsequently reported to the β-actin housekeeping protein.

##### Flow Cytometry-Based Immunoassay

To evaluate the anti-inflammatory potential of MNP-Dex/PCA, M1 polarized macrophages were incubated for 48 h with MNP-Dex/PCA containing 350 µM PCA, and relevant controls (plain MNP-Dex, free PCA, and Dexa). Then, the IL-6 cytokine level was measured in culture medium using a bead-based flow cytometry technique (Human IL-6 Flex Set Kit, BD Biosciences, San Jose, CA, USA) as previously described [29]. Samples were centrifuged at 10,000 rpm for 10 min and analyzed using the Gallios^TM^ flow cytometer (Beckman Coulter Life Sciences, Brea, CA, USA). Instrument setup was achieved using the beads from the Human Soluble Protein Master Buffer Kit (BD Biosciences, USA) according to the manufacturer’s instructions. Data processing was performed using the Kaluza 1.5 software (Beckman Coulter Life Sciences, Brea, CA, USA). IL-6 concentration (pg/mL) values were calculated using the calibration curve supplied by the kit.

### 2.5. Statistical Analysis

Statistical data analysis was performed by one-way ANOVA with Dunnett’s or Tukey post hoc test using GraphPad^TM^ Prism software version 7.03 (GraphPad Software Inc., San Diego, CA, USA). Statistically significant differences were considered for *p* values < 0.05. The results were expressed as mean ± standard deviation (S.D.) of at least two independent experiments and replicates.

## 3. Results

### 3.1. Physico-Chemical Characterization of PCA-Loaded Magnetic Nanoparticles

#### 3.1.1. Morphological Analysis of Nanoparticles by TEM

**Figure 1:**
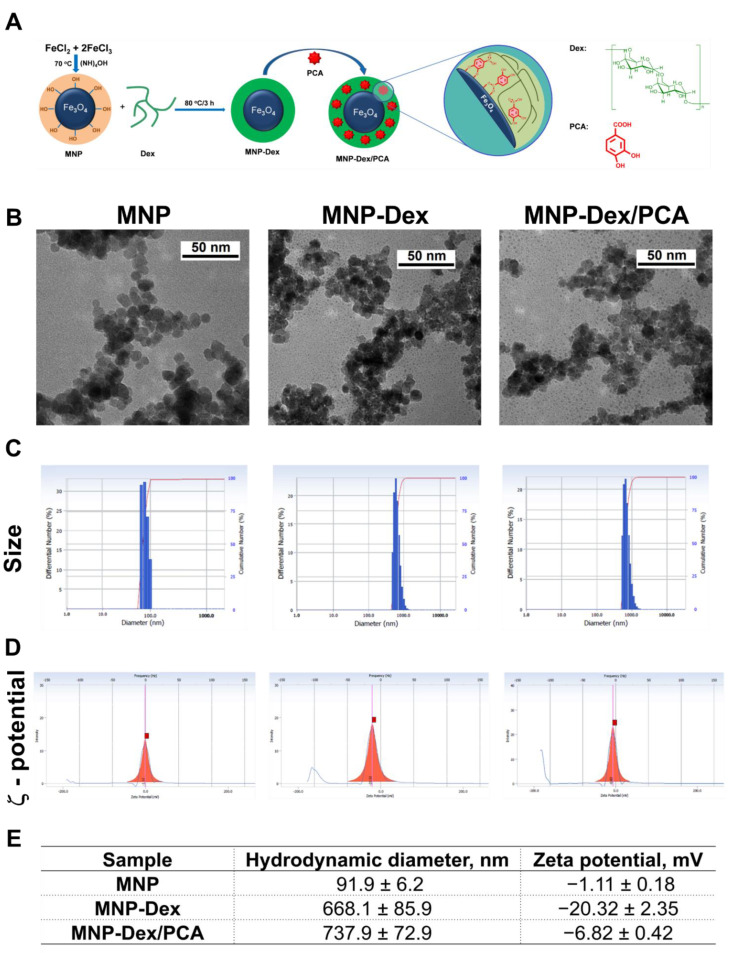
The schematic representation of MNP-Dex/PCA synthesis (**A**). Electron micrographs (**B**); representative measurements of average hydrodynamic diameter (**C**); and ζ-potential (**D**) of MNP, MNP-Dex, and MNP-Dex/PCA. Results reported as mean ± S.D. for 3 individual measurements (**E**).

The analysis of TEM images reveals nanoparticles of approximately spherical shape for MNP, MNP-Dex, and MNP-Dex/PCA, having an aggregation tendency (Figure 1A). Following the preparation processes, MNP have approximately the same shape and size as the resulting products, MNP-Dex and MNP-Dex/PCA, having a diameter in the range of 7–10 nm. Obviously, the morphology and the similar dimensions for all types of nanoparticles are due to the fact that the metal oxide core does not change after the preparation processes, and in our case, at this resolution, the TEM shows mainly the inorganic core.

#### 3.1.2. Size and Zeta Potential

The average hydrodynamic diameter and ζ-potential of magnetic nanoparticles (uncoated MNP, MNP-Dex, and MNP-Dex/PCA) were measured after 1:1000 dilution in filtered distilled water (Figure 1B–D). Dynamic light scattering revealed that the hydrodynamic diameter of nanoparticles increased after dextran coating, from 90 nm for uncoated MNP to 670 nm for MNP-Dex. A possible explanation may be the expansion of dextran due to the solvation of the polymer chains in water, which leads to a substantial increase in the hydrodynamic diameter. On the other hand, the increases in the hydrodynamic diameter may be due to the aggregation phenomena, as observed in the analysis of TEM micrographs. These results are in agreement with previous results reported for MNP coated with dextran [22], and polyethyleneimine [23,30]. Similarly, the size of MNP-Dex/PCA increased by 70 nm when compared to MNP-Dex, indicating that PCA was loaded on the surface of dextran-coated MNP (Figure 1B,D).

The measurements of ζ-potential show that it changes as a result of MNP functionalization (Figure 1C,D). Thus, in water at neutral pH, MNP has a relatively low negative ζ-potential (−1 mV), pointing out the propensity of MNP for aggregation. The covering of MNP with dextran leads to an increase in the negative value of the ζ-potential, which reaches a value of about −20 mV, indicative of the nanoparticles’ stability (nanoparticles are less likely to aggregate). The addition of PCA determines a decrease in the negative value of ζ-potential up to ~−7 mV). The inclusion of PCA in the dextran layer may conceivably cause the masking of the negative charges of dextran. This process can lead to a slight intensification in aggregation phenomena, as observed by the increase in the size, which in turn amplifies the decrease in the negative value of the zeta potential (Figure 1D).

#### 3.1.3. PCA Loading Efficiency

Following HPLC analysis, it was established that the loading efficiency of PCA is 87% (8.7 mg PCA are captured on the surface of 10 mg of MNP).

### 3.2. In Vitro Evaluation of PCA-Loaded Magnetic Nanoparticles Cytotoxicity

**Figure 2:**
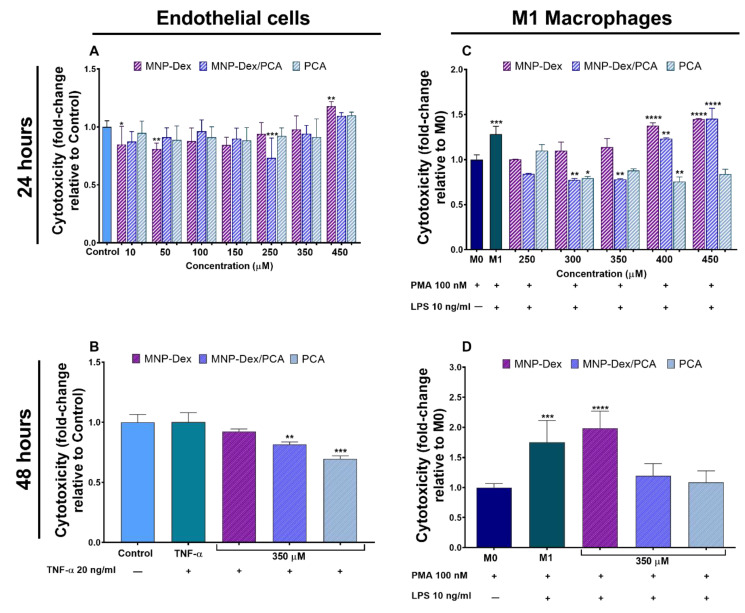
Cell cytotoxicity after 24 h of incubation with increasing concentrations of MNP-Dex, MNP-Dex/PCA, and PCA on human endothelial cells (**A**) and M1 type macrophages (**C**), and after 48 h of incubation of human endothelial cells in the absence (‒) or the presence (+) of TNF-α (**B**) and M1 macrophages (**D**) with the chosen pharmacologically relevant concentration of PCA (350 μM) loaded on MNP (MNP-Dex/PCA), plain MNP-Dex, and free PCA. * *p* < 0.05, ** *p* < 0.01, *** *p* < 0.001, and **** *p* < 0.0001 vs. control cells (EC or M0 macrophages cultured in the absence of any treatment).

The cytotoxic effect of MNP-Dex/PCA was assessed in two cell types, EA.hy926 cells and THP-1-derived macrophages, by means of two cell cytotoxicity tests: XTT (Figure 2A) and ToxiLight (Figure 2B–D) assays. Cells were treated with increasing doses of MNP-Dex/PCA, corresponding to polyphenol concentrations of 10, 50, 100, 150, 250, 350, and 450 μM (in the case of EA.hy926 cells), or 250, 300, 350, 400, and 450 μM (in the case of THP-1 derived M1 macrophages). After 24 h of treatment, it can be observed that the PCA-functionalized magnetic nanoparticles did not have a cytotoxic effect on EA.hy926 cells at any concentration tested. An increased cytotoxic effect can be observed on M1 macrophages at higher concentrations of captured PCA (400 and 450 μM) (Figure 2A,C). Concentrations equal to or exceeding 70 µg/mL plain MNP-Dex, the concentration corresponding to MNP loaded with 400 μM PCA, proved to be cytotoxic for both cell types after 24 h of incubation (Figure 2A,C). The free polyphenol did not have a cytotoxic effect on the two cell types at any concentration investigated. On the contrary, as PCA concentration increased, M1 phenotype macrophages released less quantifiable adenylate kinase in the cell medium, implying that the polyphenol could reverse the cytotoxicity induced by cell treatment with PMA and LPS, used in THP-1 monocytes differentiation. A similar effect can be observed when M1 macrophages are treated with MNP-Dex/PCA, in which polyphenol concentration was below 400 μM (Figure 2C).

The working concentration of 350 μM PCA loaded on MNP-Dex nanoparticles was chosen, based on our results showing the absence of cytotoxicity and on therapeutic properties of the polyphenol at this concentration, as previously reported [31,32,33]. Similarly, the corresponding iron concentration of MNP-Dex and MNP-Dex/PCA (i.e., 62 μg/mL), was found to be non-cytotoxic on both cell types tested as stated above.

Cell cytotoxicity was also assessed after 48 h of treatment, but only with the selected pharmacologically relevant concentration of PCA-loaded MNP and the corresponding controls (free PCA and plain MNP-Dex). MNP-Dex/PCA and free PCA did not have a cytotoxic effect on both cell types (Figure 2B,D), while MNP-Dex treatment of M1 macrophages determined an increase in cytotoxicity of approximately two-fold compared to control M0 macrophages, which can also be observed in the case of untreated M1 macrophages (Figure 2D). This result could be explained by the fact that plain MNP-Dex, unlike PCA and MNP-Dex/PCA, do not reverse the cytotoxic effect of the two inductors of monocyte to macrophage differentiation, PMA and LPS (Figure 2C,D). There were no significant differences in cell cytotoxicity between MNP-Dex-treated and untreated EC, as well as TNF-α-stimulated EC (Figure 2B).

### 3.3. Intracellular Localization of MNP-Dex/PCA

**Figure 3:**
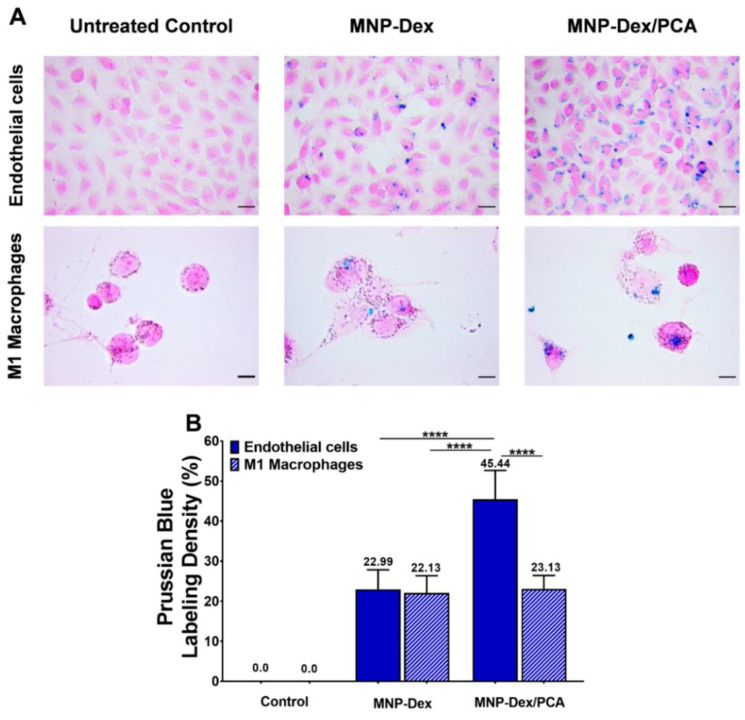
Bright-field microscopy images showing the uptake of magnetic nanoparticles at 62 μg/mL concentration in EA.hy926 cells (Endothelial cells) and THP-1 derived M1 macrophages after Prussian Blue staining (**A**) and the quantification of iron-labelled cells using ImageJ software (**B**). The data are expressed as mean ± SD from two independent experiments. **** *p* < 0.0001. Scale Bar: 10 μM.

The intracellular localization of MNP-Dex/PCA and plain MNP-Dex was assessed microscopically using the Prussian Blue staining, which is based on the formation of a blue water-insoluble complex between potassium ferrocyanide and the released ferric ions. The data obtained revealed that MNP-Dex/PCA, as well as MNP-Dex, were internalized by EA.hy926 cells and THP-1 derived M1 macrophages after 24 h of incubation (blue spots, Figure 3A). MNP-Dex were internalized at similar rate by M1 macrophages and EC, with a staining density of approximately 22% compared to untreated controls, whereas MNP-Dex/PCA was taken up nearly two-fold higher in the case of EC compared to M1 macrophages (*p* < 0.001, Figure 3B). MNP-Dex/PCA was also found to be internalized two-fold higher by EC than plain MNP-Dex, although both magnetic nanoparticles are similarly internalized by M1 macrophages (Figure 3A,B). This could imply that EC internalize polyphenol-functionalized MNP more efficiently by receptors that specifically recognize polyphenols, for example, estrogen receptors (ER) [34], which are absent on the surface of M1 macrophages [35].

### 3.4. Anti-Inflammatory Effect of MNP-Dex/PCA

#### 3.4.1. Anti-Inflammatory Activity of MNP-Dex/PCA on Activated EC

**Figure 4:**
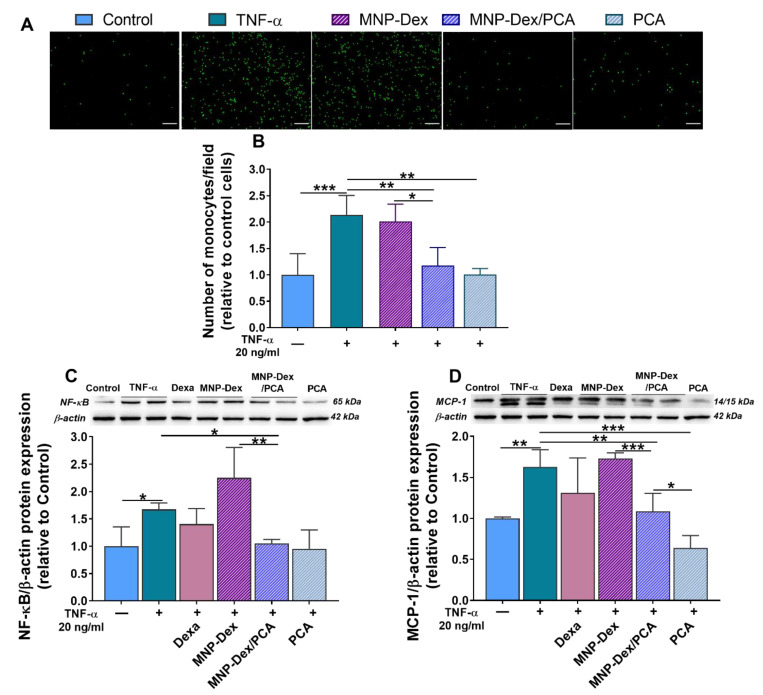
Anti-inflammatory effects of MNP-Dex/PCA on activated EC. Functional role of MNP-Dex/PCA in reducing THP-1 monocytes adhesion to TNF-α (20 ng/mL)-activated EC (+). Fluorescence microscopy images showing fluorescently labelled monocytes (green, **A**) and quantification of adhered monocytes expressed as number/field (**B**). Protein expression of NF-kB p65 (**C**) and MCP-1 (**D**) in quiescent (‒) and TNF-α activated EC (+) treated for 48 h with MNP-Dex/PCA, MNP-Dex, and free PCA at a pharmacologically relevant concentration of PCA (350 μM). Dexa: treatment of EC with dexamethasone (2.5 µM). Protein expression was normalized to β-actin. The data are expressed as mean ± SD from 3 independent experiments. * *p* < 0.05, ** *p* < 0.01, and *** *p* < 0.001.

The anti-inflammatory activity of MNP-Dex/PCA on TNF-α activated EC was validated, at a functional level, employing the monocyte adhesion assay. In addition, the protein expression of NF-κB p65 subunit and MCP-1, molecules involved in the inflammatory response, was determined in EC exposed to TNF-α and treated with MNP-Dex/PCA, MNP-Dex, or free PCA. An increase in the number of adherent monocytes to TNF-α activated EC as compared to unstimulated, control EC was measured (Figure 4A,B). This result is a consequence of the increases in NF-κB p65 and MCP-1 protein levels in TNF-α activated EC (Figure 4C,D).

We found that 48 h of treatment with both MNP-Dex/PCA and free PCA reduced the adhesion of monocytes to TNF-α activated EC (~33%, *p* < 0.01) compared with that to activated EC, left untreated, whereas plain MNP-Dex did not modify the number of adherent monocytes (Figure 4A,B). The anti-inflammatory effect of the MNP-Dex/PCA treatment was further confirmed by a decrease in the protein expression of NF-κB p65 subunit and MCP-1 (~35%, *p* < 0.01, and ~33%, *p* < 0.05, respectively), while the incubation with MNP-Dex exhibited no significant differences in protein levels in comparison to TNF-α-stimulated EC (Figure 4C,D). No significant difference in NF-kB and MCP-1 expression was observed when activated EC were treated with dexamethasone (2.5 µM).

#### 3.4.2. Anti-Inflammatory Activity of MNP-Dex/PCA on M1 Macrophages

**Figure 5:**
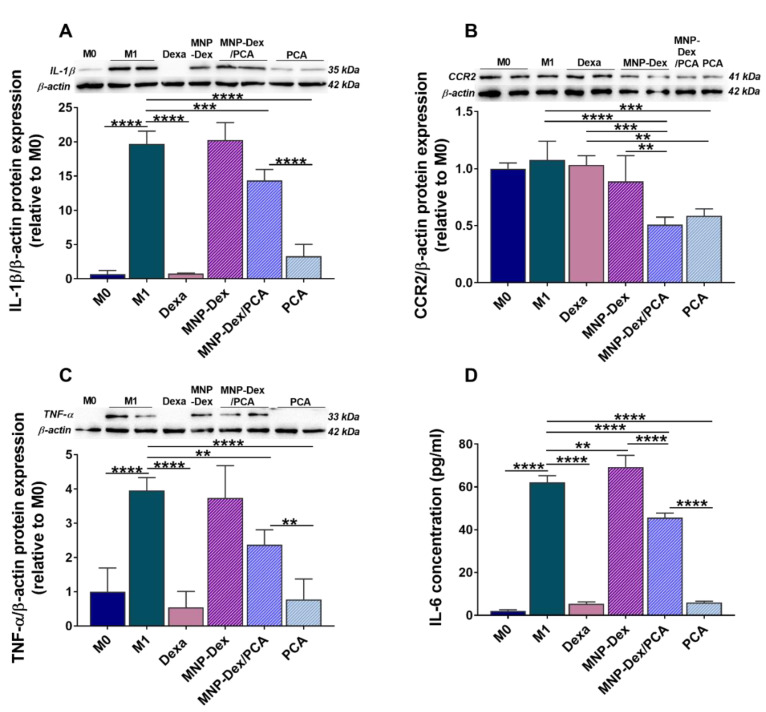
The anti-inflammatory effect of MNP-Dex/PCA on THP-1-derived M1 macrophages was determined by evaluating the expression of intracellular pro-inflammatory molecules IL-1β (**A**), CCR2 (**B**), TNF-α (**C**), and the levels of released IL-6 (**D**) after 48 h of treatment. Protein expression was normalized to β-actin. For comparison, the levels of pro-inflammatory molecules in M0 macrophages were shown. The data are expressed as mean ± SD from at least three independent experiments performed in duplicates. ** *p* < 0.01, *** *p* < 0.001, and **** *p* < 0.0001. The protein levels of cytokines in M0 macrophages are also shown. Dexa-treatment of M1 macrophages with dexamethasone (2.5 µM).

The expression of pro-inflammatory cytokines IL-1β; TNF-α; and that of the receptor for MCP-1 chemokine, CCR2, were evaluated by Western Blotting in M1 macrophages after incubation, for 48 h, with MNP-Dex/PCA and the corresponding concentrations of plain MNP-Dex and free PCA. As controls, untreated and dexamethasone-treated M1 macrophages were used. THP-1-derived M1 macrophages expressed significantly higher levels of IL-1β, TNF-α, and IL-6 compared to M0 macrophages, indicating the successful polarization of the cells towards a pro-inflammatory phenotype (Figure 5). The data shows a similar decrease pattern in the levels of the four pro-inflammatory molecules investigated after incubating the cells with MNP-Dex/PCA. Thus, MNP-Dex/PCA treatment reduced the protein expression of IL-1β by ~27% (*p* < 0.001), CCR2 by ~55% (*p* < 0.0001), TNF-α by ~40% (*p* < 0.01), and IL-6 by ~27% (*p* < 0.0001) in M1 macrophages in comparison to untreated ones (Figure 5A–D). The free PCA also reduced the levels of the pro-inflammatory molecules and in a more drastic manner in the case of IL-1β (~83%, *p* > 0.0001), TNF-α (~80%, *p* > 0.001), and IL-6 (90%, *p* > 0.0001), which is similar to the effect of Dexa (Figure 5A–D). On the other hand, plain MNP-Dex-treated M1 macrophages had no significant changes in the protein expression of IL-1β, CCR2, and TNF-α, while IL-6 concentration increased by ~11% (*p* < 0.01). Dexamethasone was used as a gold standard anti-inflammatory drug.

#### 3.4.3. The Effect of MNP-Dex/PCA on Key Proteins of MAPKs Pathway

**Figure 6:**
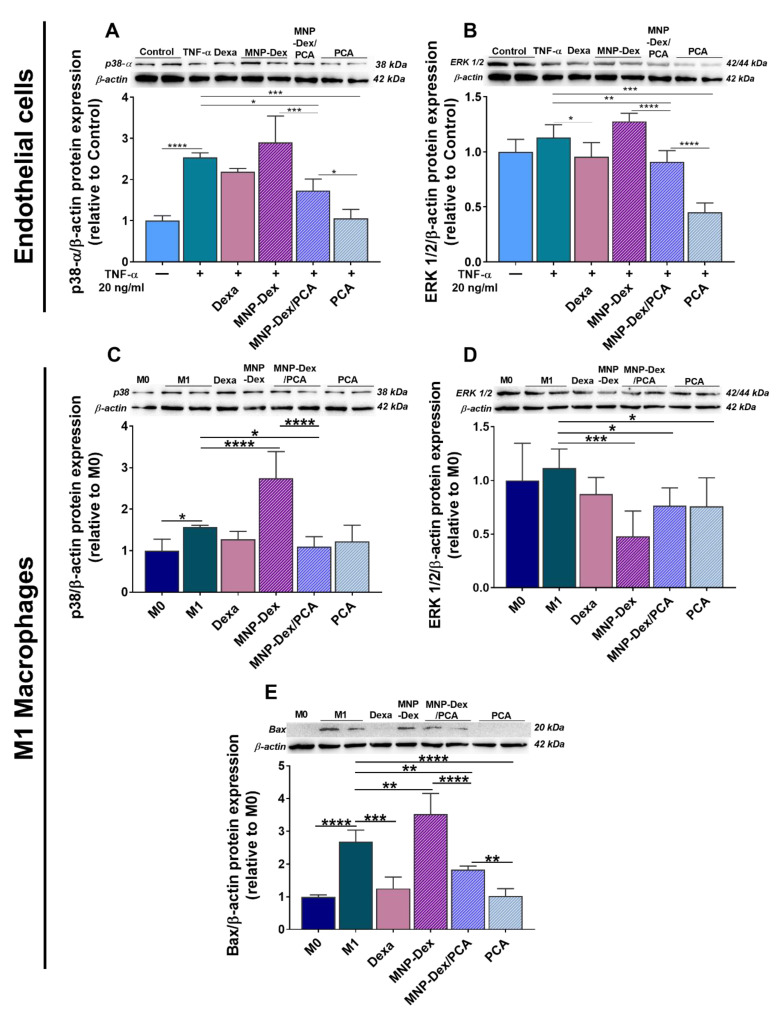
Protein expression of p38-α (**A**,**C**) and total ERK 1/2 (**B**,**D**) in quiescent (‒) and TNF-α (20 ng/mL)-activated (+) EA.hy926 cells and M1 macrophages after 48 h of incubation with MNP-Dex/PCA (62 µg/mL MNP/ 350 µM PCA), as well as the protein expression of anti-apoptotic molecule Bax in M1 macrophages (**E**). Plain MNP-Dex, free PCA, and dexamethasone (Dexa) were used as controls. Protein expression was normalized to β-actin. The data are expressed as mean ± SD from three independent experiments. * *p* < 0.05, ** *p* < 0.01, *** *p* < 0.001, and **** *p* < 0.0001.

The protein expression of pro-inflammatory molecules can be increased by the activation of specific signaling components of the MAPKs pathway. To further investigate the anti-inflammatory activity of MNP-Dex/PCA, we assessed their effect on two main molecules of the MAPK signaling pathway: p38-α and ERK 1/2, and compared it with that of MNP-Dex, free PCA, and the well-known anti-inflammatory agent dexamethasone (Figure 6). At the concentration tested of 2.5 µM dexamethasone, the data revealed little or no significant differences in the protein levels of p38-α and ERK 1/2 when compared to TNF-α activated EC and PMA/LPS-activated M1 macrophages. On the other hand, the MNP-Dex/PCA treatment significantly decreased p38-α protein expression in both cell types (~32% in EA.hy926 cells and ~30% in M1 macrophages, *p* < 0.05) (Figure 6A,C) compared to the stimulated control cells (TNF-α activated EA.hy926 cells and PMA/LPS activated M1 macrophages). The treatment with plain MNP increased the synthesis of p38-α isoform (~75%, *p* < 0.0001) in M1 macrophages (Figure 6C).

ERK1/2 protein synthesis exhibited no significant differences when TNF-α-stimulated EA.hy926 cells were treated with MNP-Dex but decreased when EC were treated with MNP-Dex/PCA (~22%, *p* < 0.01) and PCA (~62%, *p* < 0.001), compared to TNF-α-stimulated EA.hy926 cells (Figure 6B). Interestingly, we found that in the case of M1 macrophages the ERK1/2 protein expression was reduced by ~57% (*p* < 0.001) after 48 h of incubation with plain MNP-Dex (Figure 6D). Because ERK1/2 is a protein involved in cell survival and proliferation, we further looked at the expression of the pro-apoptotic molecule B-cell lymphoma 2 (Bcl-2)-associated X protein (Bax) in M1 macrophages treated with MNP, MNP-Dex/PCA, and free PCA compared to M0 and M1 untreated macrophages (Figure 6E). Thus, we found that Bax level was significantly enhanced (~31%, *p* < 0.01) upon MNP-Dex treatment, while MNP-Dex/PCA, free PCA, and Dexa reduced Bax level by ~32% (*p* < 0.01), ~62% (*p* < 0.0001), and ~50% (*p* < 0.001), respectively, when compared to untreated M1 macrophages, suggesting an anti-apoptotic effect of these three formulations. This result may be a possible explanation of the decrease in ERK1/2 protein expression in M1 macrophages treated with plain MNP-Dex.

## 4. Discussion

Inflammation is a process involved in the inception and progression of atherosclerosis (AS), triggered by endothelial activation and characterized by an increase in the expression of cell adhesion molecules (CAM) and chemokines, facilitating the recruitment and transmigration of immune cells [36,37]. The key role played by the inflammatory molecules such as cytokines and chemokines in the initiation and progression of AS has been well established. Many drug therapies have been developed to prevent or reduce the formation of atherosclerotic plaques; however, the mortality rate from CVD is increasing alarmingly [1]. Thus, targeting chronic inflammation with potent inhibitors of chemokines and cytokines expression is critical, requiring new and innovative nanotherapeutic strategies.

Because many health-beneficial properties of polyphenols have been described, including anti-inflammatory activities, attempts have been made to introduce them as novel complementary drugs in the prevention and/or treatment of non-communicable diseases having an associated inflammatory process, including CVD, diabetes, and cancer [38]. Although in vitro studies have confirmed protocatechuic acid (PCA)’s therapeutic potential in alleviating exacerbated inflammatory processes [32,33,39], the reduced bioavailability prevents the exercising of the therapeutic properties in vivo [40,41]. Thus, the formulation into nanoparticles may represent a good strategy to increase its concentration at the affected sites.

Since EC and macrophages are key players in the onset and progression of chronic vascular inflammation, the focus of this paper was to synthesize, characterize, and further investigate the anti-inflammatory potential of novel PCA-functionalized dextran-coated magnetic nanoparticles (MNP-Dex/PCA) on these cells by examining the protein expression of various pro-inflammatory molecules (e.g., IL-1β, TNF-α, IL-6, MCP-1, and CCR2) and signaling molecules involved in inflammation (e.g., NF-kB, p38-α, and ERK 1/2). Moreover, the functional role of MNP-Dex/PCA in inhibiting monocyte adhesion to activated EC was investigated. We developed a nanocarrier with a core shell-like structure consisting of an iron oxide hydrophobic core and a hydrophilic shell represented by the polysaccharide dextran (MNP-Dex), which serves as a stabilizer. The tendency to aggregation of uncoated MNP decreases after their coating with dextran, as suggested by the higher negative value of the ζ-potential, whereas the higher hydrodynamic diameter is due to the expansion of the coating polymer in water. The loading of PCA on the surface of MNP-Dex results in an increase of the hydrodynamic diameter and a decrease in the ζ-potential negative value. The dextran coating and PCA loading of magnetic nanoparticles cause minor changes from a morphological point of view, as proved by the TEM investigation.

According to in vivo studies, iron oxide nanoparticles are relatively safe because they do not accumulate in vital organs and are quickly removed from the body [42]. Dextran is a polymer used in various applications where the surface of material needs to be modified to improve its properties [43]. The presence of dextran on its surface has been shown to attenuate iron oxide toxicity in EC and monocytes [44,45]. In vitro cytotoxicity evaluation after 24 h showed that MNP-Dex/PCA possess good biocompatibility at iron concentrations less than 100 µg/mL. Our findings are consistent with previously published data indicating that nanoparticles with negative or slightly negative zeta potential are less likely to be cytotoxic and show good internalization [46,47,48]. The polyphenol used in this study (PCA) was also found to be non-cytotoxic at all concentrations tested. We further investigated the cytotoxicity of MNP-Dex/PCA after 48 h of incubation with the pharmacologically relevant concentration of PCA on EC and M1 type macrophages, as well as the corresponding controls. While MNP-Dex/PCA and free PCA (350 µM) had no cytotoxic effect on both cell types, plain MNP-Dex increased the cell cytotoxicity of M1 macrophages by almost two-fold, which could be attributed to LPS stimulation, and a similar cytotoxicity was also observed in untreated M1 macrophages.

The qualitative Prussian Blue staining confirms the internalization of nanoparticles by both EC and M1 macrophages. While plain MNP-Dex and MNP-Dex/PCA are taken up at the same rate by M1 macrophages, the data showed higher uptake in the case of EC when PCA is loaded on the surface of MNP-Dex, which is consistent with recent findings highlighting polyphenols’ ability to interact with the lipid bilayer, as well as specific membrane receptors, which could ultimately facilitate nanoparticle-uptake [49,50]. As a result, we expect that the increased uptake of MNP-Dex/PCA by EC after 24 h would determine maximum targeting efficacy when targeting the vascular wall. It was also revealed that, in the case of dietary iron, polyphenol addition increases uptake in human colorectal adenocarcinoma (Caco-2) epithelial cells [51]. Nevertheless, there is little information about the role of transporters in cellular accumulation, and evidence for individual phenolic compounds is limited.

Previous studies demonstrated the ability of PCA to inhibit monocyte adhesion to the mouse aortic endothelial cells in vitro and in vivo by reducing the expression of CAM such as ICAM-1 and VCAM-1, both as a consequence of inhibiting the activity of the transcription factor NF-kB [52]. There was also evidence of PCA’s anti-inflammatory effect on different types of cultured macrophages [53,54] as well as isolated from in vivo sources [55]. In the current study, we found that MNP-Dex/PCA exerts its anti-inflammatory activity on human EC and THP-1-derived M1 macrophages by lowering the levels of essential pro-inflammatory and signaling molecules involved in the cellular inflammatory process (e.g., IL-1β, TNF-α, IL-6, MCP-1, CCR2 and NF-κB, p38-α, and ERK 1/2, respectively). As a result, unlike the plain MNP, the treatment of EC with MNP-Dex/PCA treatment significantly reduced the number of adhered monocytes on EC, as well as NF-kB and MCP-1 protein expression. In the case of M1 macrophages, MNP-Dex/PCA determined not only a reduction in IL-1β, TNF-α, and CCR2 protein expression but also a decrease in the concentration of IL-6 released in the culture medium. In contrast, the data suggest that plain MNP-Dex have slightly pro-inflammatory properties on macrophages, aggravating the already LPS-stimulated cells. This action is countered by the presence of PCA on the surface of MNP, which is consistent with published data highlighting the protective effect of PCA [56,57].

Interestingly, while the well-known anti-inflammatory drug dexamethasone, which was used as a positive control, had no significant anti-inflammatory effect on activated EC at the tested concentration (2.5 µM), it significantly reduces the expression of IL-1β and TNF-α, as well as the extracellular releasing of IL-6 by M1 macrophages. This result could imply that a concentration higher than 2.5 µM is required to produce an effect on EC. Similarly, Nehme and Edelman (2008) found that Dexa did not suppress IL-1β and TNF-α in retinal EC, pointing to a possible mechanism of resistance [58].

The ability of cells to induce the over-expression of inflammatory mediators is a primary biological response to the activation of NF-kB and MAPK pathways. NF-kB is a recognized regulator of inflammation that controls, in EC, the expression of MCP-1, a chemokine involved in monocytes recruitment and activation, and in macrophages, the production of cytokines such as IL-1β, TNF-α, and IL-6 [9,10]. The isoform p38-α-MAPK, in particular, is expressed in both EC and macrophages and plays a vital role in regulating cellular processes involved in inflammation [59]. ERK 1/2 also regulates cellular differentiation, proliferation, and survival, as well as inflammatory processes [60]. We were interested in how MNP-Dex/PCA affected the protein expression of these signaling molecules, given that it is known to be responsible for the over-expression of inflammatory molecules. We found that MNP-Dex/PCA treatment reduced NF-kB expression in EC, as well as p38-α synthesis in both cell types, supporting the anti-inflammatory activity reported for PCA. In contrast, plain MNP did not change the level of p38-α in TNF-α‒activated EC but increased p38-α protein synthesis in M1 macrophages. Others have thoroughly documented MNP’s pro-inflammatory effect, which has been attributed to consistent macrophage reactive oxygen species (ROS) production as well as an iron overload, both of which have been shown to aggravate inflammation in LPS-stimulated mice [61,62]. MNP-Dex/PCA inhibited ERK protein synthesis in both cell types, similar to the effect seen with p38-α, and the reduction was significantly higher when M1 macrophages were treated with plain MNP. The discrepancy could be attributed to ERK’s role in cellular proliferation and survival, with its expression being reduced as a result of the increase in cell cytotoxicity and increase in pro-apoptotic Bax protein expression observed after 48 h of M1 macrophages incubation with plain MNP.

## 5. Conclusions

In this study, new biocompatible and non-cytotoxic dextran-coated iron oxide nanoparticles for the delivery of the protocatechuic acid were synthesized. We demonstrated that (i) MNP-Dex/PCA reduce the adhesion of monocytes to TNF-α-activated EC; (ii) MNP-Dex/PCA have anti-inflammatory activity at non-cytotoxic concentrations by reducing the levels of pro-inflammatory mediators such as MCP-1, IL-1β, TNF-α, IL-6, and CCR2 in activated EC and M1 phenotype macrophages; and (iii) the anti-inflammatory effect of MNP-Dex/PCA was associated with the reduction in the levels of p38-α and ERK1/2 MAPKs and NF-kB transcription factor.

The benefit to formulate PCA into MNP-Dex nanoparticles is that we developed a theranostic system able to guide the therapeutic agent to the target due to the magnetic properties of the nanocarrier and to potentiate the efficiency of PCA as an anti-inflammatory agent by improving its cellular internalization.

According to our findings, the newly developed dextran shell-iron oxide core nanocarriers could be used as theranostic tools for guidance, imaging, and therapy of various inflammatory diseases, including atherosclerosis.
